# Predictors of quality of life among youths in foster care—a 5-year prospective follow-up study

**DOI:** 10.1007/s11136-020-02641-z

**Published:** 2020-09-24

**Authors:** Marit Larsen, Anouk Goemans, Valborg Baste, Tom F. Wilderjans, Stine Lehmann

**Affiliations:** 1 Regional Centre for Child and Youth Mental Health and Child Welfare, NORCE Norwegian Research Centre, Nygårdstangen, Postbox 22, 5838 Bergen, Norway; 2 Institute of Education and Child Studies, Leiden University, Leiden, The Netherlands; 3 NORCE Norwegian Research Centre, Bergen, Norway; 4 Methodology and Statistics Research Unit, Institute of Psychology, Faculty of Social and Behavioural Sciences, Leiden University, Pieter de la Court Building, Wassenaarseweg 52, 2333 AK Leiden, The Netherlands; 5 Research Group of Quantitative Psychology and Individual Differences, Faculty of Psychology and Educational Sciences, KU Leuven, Tiensestraat 102, Box 3713, 3000 Leuven, Belgium; 6 Leiden Institute for Brain and Cognition (LIBC), Leids Universitair Medisch Centrum (LUMC), 2300 RC Leiden, The Netherlands; 7 Department of Health Promotion and Development, Faculty of Psychology, University of Bergen, Bergen, Norway

**Keywords:** Quality of life, QoL, Foster care, Youth, Predictors, Prospective study

## Abstract

**Purpose:**

Few studies have investigated possible predictors of positive outcomes for youths in foster care. The aim of this prospective follow-up study was to examine quality of life (QoL) among youths in foster care and to assess whether contextual and child factors predicted QoL.

**Methods:**

Online questionnaires were completed by carers in Norway in 2012 (T1, *n* = 236, child age 6–12 years) and by youths and carers in 2017 (T2, *n* = 405, youth age 11–18 years). We received responses on 116 of the youths at both T1 and T2, and our final sample consisted of 525 youths with responses from T1 and/or T2. Child welfare caseworkers reported preplacement maltreatment and service use at T1. We assessed mental health and prosocial behavior at T1 by having carers complete the Strength and Difficulties Questionnaire and QoL at T2 with youth-reported KIDSCREEN-27. We analyzed the data using descriptive statistics, *t*-tests and multiple linear regressions, and we used multiple imputation to handle missing data.

**Results:**

Youths in foster care had lower QoL across all dimensions compared to a Swedish general youth sample. QoL scores among our sample were similar to Norwegian youths with ill or substance abusing parents and to European norm data. Youths reported the highest QoL scores on the parent relations and autonomy dimension. Male gender, younger age, kinship care and prosocial behavior five years earlier predicted higher QoL.

**Conclusion:**

Similar to other at-risk youths, youths in foster care seem to have lower QoL than the general Scandinavian population. Despite early adversities, they had good relations with their current carers. Adolescent girls seem especially vulnerable to low QoL and might need extra support to have good lives in foster care.

**Electronic supplementary material:**

The online version of this article (10.1007/s11136-020-02641-z) contains supplementary material, which is available to authorized users.

## Introduction

While a high prevalence of mental and physical health problems among youths in foster care is well documented [1, 2], less is known about youths in foster care that have good lives. Studies following youths in foster care over time are needed to identify predictors of positive outcomes [3, 4]. Our study examined quality of life (QoL) among youths in foster care and compared them to other youth populations. Furthermore, we investigated whether contextual and child factors were predictive of QoL in adolescence.

QoL is a multidimensional construct that encompasses physical, emotional, mental, social and behavioral components of wellbeing and functioning as perceived by the individual [5]. As QoL is a subjective experience, the gold standard of assessment is self-report [6]. While research on QoL among youths in care is scarce, findings across countries suggest that youths in foster care [7] and youths in residential care [8–11] have a poorer QoL or health related quality of life (HRQoL) than youths in the general population (see Supplementary material 1 for an overview of studies on QoL and related terms cited in this introduction). However, some European studies found similar HRQoL [10] and subjective wellbeing [12] scores for youths in foster care and youths in the general population. Furthermore, youths in foster care report higher HRQoL [13], higher subjective wellbeing [12, 14], and more positive perceptions of their care situation [15] than youths in residential care. Thus, the current knowledge suggests that youths in foster care have a higher QoL than youths in residential care, but it is unclear whether they have lower QoL than youths in the general population. To gain knowledge about how foster care-related experiences specifically affect QoL, we need more studies comparing QoL between youths in foster care and youths reared in their family of origin.

Even though information about predictors of high QoL is crucial for helping more youths having a good life in foster care, there is a lack of studies following youths in foster care over time examining QoL. However, some cross-sectional studies exist that provide information about factors associated with QoL, HRQoL or subjective wellbeing, which makes them relevant to study as possible predictors. Across European countries, boys report higher QoL and HRQoL than girls in the general population [16, 17], and among youths in care [10, 18]. Furthermore, younger children report higher QoL and subjective wellbeing than adolescents in the general European population [17], and in care [14]. However, some studies of youths in care found no relation between HRQoL and gender [7] or age [7, 19], indicating that it is unclear how gender and age are related to QoL in this population.

Although findings are mixed [20], in general, maltreated children report lower HRQoL than children in the general population [21], and exposure to maltreatment is associated with lower QoL and HRQoL among youths in the general population [16, 22] and in residential care [6, 9]. Among young people in protective custody, experiences of family violence were related to lower HRQoL, while family instability (i.e., parental drug use, mental health problems and/or absent parents) was not [23]. These findings suggest that violent experiences may be especially relevant to study as a predictor of QoL among youths in care.

A positive association between placement stability and subjective wellbeing has been found among youths in care [14]. Furthermore, a Cochrane review indicated that youths in kinship care had higher wellbeing compared to youths in nonkinship foster care [24]. However, other studies found no association between HRQoL and the number of earlier placements [7] or the age of entry into care [23]. Thus, the relationship between placement characteristics and QoL is unclear.

Youths in contact with health care professionals had poorer QoL than youths without health care contact [25]. Youths in foster care have extensive service contact [26, 27], but there is a lack of studies investigating the relationship between service use and QoL for this group. Studies indicate that mental health problems were associated with low QoL and HRQoL among youth in care [8, 13, 18]. Good interpersonal relationships, however, contributed to subjective wellbeing among youths in care [14]. Overall, the findings indicate that service contact, mental health and social relationships might predict QoL among youths in foster care.

As most studies are cross-sectional, there is a need for studies following youths in foster care over time to identify predictors of QoL. This knowledge is necessary to inform services and informal networks about areas to focus on to enhance the wellbeing and positive development of youths in foster care. Furthermore, such knowledge may inform child welfare services (CWS) about how to organize placements to enable good lives. In addition, this information can benefit the whole population of youths in foster care, not only those with mental health problems. Lastly, there are substantial differences in how the child protective services are organized in different countries [28]. Therefore, it is uncertain how transferable the knowledge about QoL among youths in foster care is between countries. Moreover, we lack studies describing the QoL of youths in foster care from the Scandinavian setting.

The first aim of the current study was to examine QoL and its subdimensions among youths in foster care in Norway and to compare their QoL scores with the scores of youths with ill or substance abusing parents, Swedish youth, and European youth. Our second aim was to examine whether QoL of youths in foster care can be predicted by contextual factors (i.e., preplacement maltreatment, kin or nonkin foster care, years in current foster home and former service contact) and child factors (i.e., mental health problems, functional impairment, and prosocial behavior) when adjusted for gender and age.

## Methods

### Procedure and study sample

**Fig. 1:**
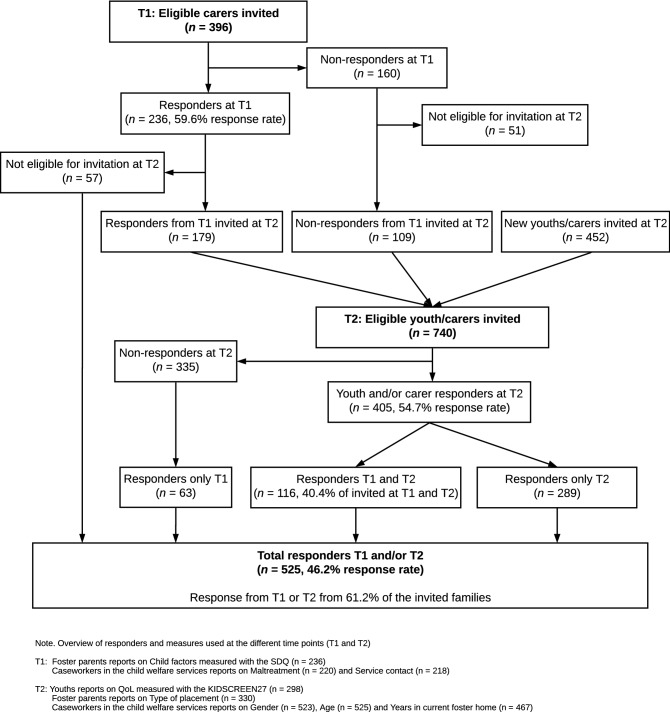
Flowchart of the data collection

The study sample is part of the research project “Young in Foster Care”, where data were collected in two waves: wave one was between September 2011 and February 2012 (T1), and wave two was between October 2016 and March 2017 (T2). Eligible participants were youths in foster care born between 1999 and 2005 who were in a legally mandated placement in the Southeast of Norway and had lived in their current foster home for at least six months. The number of eligible participants was 396 at T1 and 740 at T2. Figure 1 provides a flowchart illustrating the data collection.

At T1, carers were invited to participate, while carers and youths were invited to participate at T2. We recruited participants via postal mail with an information letter describing the study and how to complete the survey, either online on a secure webpage or by telephone interview. We asked foster mothers, foster fathers and youths to complete the survey separately. We provided reminders by post and subsequent telephone contact. We compensated youths with a gift card of 300 NOK (approximately 38 USD) for participating. Carers were not compensated.

In this study, we included all youths who had a response from a carer at T1 (*n* = 236) and/or a response from the youth (*n* = 303) and/or carer (*n* = 330) at T2 (total *n* T2 = 405). As we have T1 and T2 responses on 116 of the youths (i.e., 40.4% of the families invited at both T1 and T2), our finale sample consisted of 525 youths in foster care (46.2% response rate; Fig. 1). We combined foster mothers (*N*_T1_ = 212, *N*_T2_ = 285) and fathers (*N*_T1_ = 106, *N*_T2_ = 120) into one group of informants for each wave. We used responses from foster fathers when the foster mother was a nonresponder; otherwise, we used responses from foster mothers.

### Ethics

The Regional Committee for Medical and Health Research Ethics, Western Norway approved the study. The Norwegian Directorate for Children, Youth and Family Affairs provided exemptions from confidentiality for caseworkers and carers. In accordance with the Norwegian ethics requirement, oral consent is required from children aged 12 years or older. We described this in the invitation letter to youths and carers, and the youths were instructed that they could inform their carers if they did not want them to participate in the study.

### Measures

#### Demographic and contextual factors

We collected information about gender, age and years in the current foster home from municipal CWS offices. We defined placement type as kinship care when carers at T2 were biologically related to their foster child. Preplacement maltreatment was measured at T1 with four custom-made yes/no questions asking the child’s caseworker in CWS if the child had been exposed to or witnessed physical or emotional (i.e., threats, verbal punishment, harsh criticism or hostility) violence in the biological family before placement. We summed these variables into a new variable called “maltreatment”, with scores ranging from 0 to 4.

Service contact was measured at T1 with three custom-made yes/no questions asking caseworkers if the child had ever been assessed by child and adolescent mental health services, educational psychological services, or habilitation services. We summed these questions into a new variable called “service contact”, with scores ranging from 0 to 3. We computed a sum score for youths when CWS had provided information about at least one of the services, and missing information on one or two providers was treated as 0 (i.e., no service contact).

#### Child factors

We measured child factors at T1 by having carers complete the Strengths and Difficulties Questionnaire (SDQ) [29]. This is a 25-item questionnaire consisting of five subscales, with five items on each subscale, assessing symptoms and impairments in the daily life of youths aged 4–17 years old. Each item is rated on a three-point scale ranging from 0–2. Four of the subscales measure symptoms and one subscale measures prosocial behavior (score range 0–10). A total difficulties score (range 0–40) is calculated by summing the symptom subscales. In addition, the SDQ contains an impact scale (range 0–10), referred to as “functional impairment”, that measures distress and interference of symptoms in the youth’s daily life [29]. The SDQ has shown satisfactory reliability and validity in general child populations [29, 30] and the predictive value of the SDQ completed by carers is supported for foster children [31]. In the current study, Cronbach’s alpha for the total and subscales ranged from good to very good, as reported in Table 1.

**Table 1:** Distribution of youth characteristics, contextual and child factors, and quality of life (QoL) among youths in foster care (*N* = 525)

	*N*	% Missing^a^	*n*	%	Mean	*SD*	Min	Max	Cronbach’s alpha
Gender—boys	523	0.4	285	54.5					
Age (in years)	525	0			14.61	2.01	11.00	17.99	
Type of placement—Kinship care	330	37.1	50	15.2					
Years in current foster home	467	11.0			7.08	4.40	0.72	17.75	
Maltreatment at T1	220	58.1			0.88	1.23	0	4	
Physical violence	220		29	13.2					
Witnessed physical violence	220		59	26.8					
Emotional abuse	220		38	17.3					
Witnessed emotional abuse	220		67	30.5					
Service contact at T1	218	58.5			1.06	0.95	0	3	
CAMHS	209		96	45.9					
Education psychology service	205		108	52.7					
Habilitation service	192		26	13.5					
Foster parent reported child factors at T1									
Total difficulties	236	55.0			15.24	7.94	0	35	.88
Prosocial behavior	236	55.0			6.84	2.30	0	10	.77
Functional impairment	236	55.0			2.69	2.74	0	10	.80
T-scores of the QoL dimensions									
General QoL	298	43.2			47.99	11.82	15.88	83.81	.87
Physical wellbeing	298	43.2			46.30	13.21	20.70	73.20	.87
Psychological wellbeing	297	43.4			49.33	13.70	17.56	73.53	.92
Parent relations and autonomy	297	43.4			52.80	13.01	1.75	74.39	.89
Social support and peers	296	43.6			50.22	11.63	23.62	66.34	.86
School environment	298	43.2			48.35	11.98	16.28	71.00	.88

^a^% missing’s are provided for the variables used in further analyses

#### QoL

We measured QoL at T2 with the KIDSCREEN-27 Quality of Life Questionnaire [25] a 27-item self-reported measure to assess five dimensions of QoL within the last week for youth aged 8–18 years old. The dimensions are physical wellbeing (e.g., *Have you felt fit and well?)*, psychological wellbeing (e.g., *Have you felt sad?)*, parent relations & autonomy (e.g., *Have your parent(s) treated you fairly?)*, peers & social support (e.g., *Have you had fun with your friends?)*, and school environment (e.g., *Have you been able to pay attention*?). Each item is scored on a five-point Likert scale (1 = ”never” or “not at all” to 5 = ”always” or “extremely”). The KIDSCREEN-10 questionnaire is embedded within the KIDSCREEN-27 questionnaire and consists of ten items that provide a single index of general QoL. In this study we utilized both the five dimensions of QoL from the KIDSCREEN-27 and the general QoL index calculated from the ten items in the KIDSCREEN-10 questionnaire. The reliability, discriminatory power, and validity of both instruments have been shown to be good [17, 25, 32]. The Norwegian version of the KIDSCREEN has shown good validity and reliability in the general population and in clinical samples [33]. Cronbach’s alpha for both instruments in the current study ranged from good to very good (Table 1). For both measures, the raw scores were computed into t-scores using a mean of 50 and a standard deviation of 10 (i.e., the mean and SD of the norm population), adopting the scoring algorithms provided by the KIDSCREEN group [25]. We applied confirmatory factor analysis (CFA) to test whether the established five-factor structure of KIDSCREEN-27 fitted our data. The five-factor structure showed an acceptable fit to our data (CFI = 0.88, RMSEA = 0.09) and was improved (CFI = 0.90, RMSEA = 0.08) by allowing items six and seven of the parent relations and autonomy dimension to correlate.

### Analyses

Descriptive statistics for gender, age (at T2), contextual and child factors, and QoL are presented as percentages, means (M), standard deviations (SD), minimum and maximum values. We compared the T1 values of gender, age, maltreatment, service use, and child factors between T1-only responders and responders at both T1 and T2 using two-sample t-tests, and found no differences between the groups. We examined the correlations between general QoL, the five QoL dimensions, and all predictors. No problems with multicollinearity were indicated between variables included in the same analysis, with functional impairment and total difficulties having the highest correlation (0.73).

We conducted two-sample *t*-tests to compare the *t*-scores on the five dimensions of QoL against the *t*-scores in a Swedish general population sample [34], a Norwegian sample of youths with ill or substance abusing parents (at-risk Norwegian youths [35]) and European norm data from youths aged 12–18 [25]. We used the same test to compare the general QoL scores in our sample to Swedish [32] and European norm data [25]. We calculated the Cohen’s *d* effect sizes of the differences between the groups by dividing the mean difference by the pooled standard deviation, where *d* = 0.2 can be considered a ‘small’ effect size, *d* = 0.5 a ‘medium’ effect size and *d* = 0.8 a ‘large’ effect size [36].

To examine possible predictors of QoL, we conducted separate linear regression analyses for general QoL and the five QoL dimensions. In each regression analysis, we added the predictors stepwise. The covariates gender and age were added first. Second, contextual factors were added (i.e., maltreatment, service contact, type of placement and years in current foster home). Last, the child factors (i.e., total difficulties, prosocial behavior, and functional impairment) were added to the model. We used multiple imputation to handle missing data. Multiple imputation models were fitted separately for general QoL and the five QoL dimensions and included all predictors from the full regression model. We imputed missing values on both predictor and outcome variables. In both imputation models, we used the sum scores of the variables, created 30 imputed datasets and pooled the results from the regression analyses into overall estimates. To investigate the effect of the missing data on the obtained results, the regression models were also fit with full information maximum likelihood (FIML) to address missing data. These additional analyses yielded similar results (see Supplementary Tables 1 and 2) which supports the validity of our findings.

Descriptive statistics were calculated using IBM SPSS Statistics 24 [37]. We conducted multiple linear regressions in R [38], and multiple imputation models were fitted with the MICE package [39].We also performed the CFA and regression analyses with FIML in R using the Lavaan package [40]. The significance level was set to 0.05.

## Results

As can be seen in Table 1, our sample consisted of 54.5% (*n* = 285, total *n* = 523) boys and had a mean age of 14.61 (SD = 2.01). On average, they had lived 7.08 years (SD = 4.40) in their current foster home, and 15.2% (*n* = 50, total *n* = 330) lived in kinship care. The foster youths had experienced, on average, less than one (*M* = 0.88, SD = 1.23) type of maltreatment with witnessing emotional abuse as the most common type (30.5%, *n* = 67, total *n* = 220). Most youths had been in contact with one service at T1 (*M* = 1.06, SD = 0.95). The mean reported total difficulties at T1 was 15.24 (SD = 7.94), and 58.9% (*n* = 139, total *n* = 236) of the responders scored at or above the suggested cut off score of 13 [31] for being in the clinical range of mental health problems for this group.

### QoL and comparison of scores to other youth samples

General QoL had high correlations to the five QoL dimensions, and the highest was with psychological wellbeing (0.85; Supplementary Table 3).The highest QoL scores were reported on the parent relations and autonomy dimension (*M* = 52.8), while the lowest scores were on physical wellbeing (*M* = 46.3; Table 2). Compared to the Swedish general youth population, the youths in our sample had lower general QoL (*d* = -0.36, *p* < 0.001) and lower scores on all QoL dimensions with small or medium effect sizes. Compared to the at-risk Norwegian youths, the youths in foster care reported lower scores on the school environment dimension (*d* = − 0.23, *p* = 0.009) but higher scores on the parent relations and autonomy dimension (*d* = 0.18, *p* = 0.041). Compared to European norm data, the youths in our sample had higher scores on the parent relations and autonomy dimension (*d* = 0.29, *p* < 0.001) but lower physical wellbeing (*d* = − 0.20, *p* < 0.001). The effect sizes of the differences between our sample and the Norwegian at-risk youths and European norm data were small.

**Table 2:** Quality of life (QoL) *t*-scores of foster youth compared to other youth groups using the KIDSCREEN-10 and KIDSCREEN-27

	Our sample			Swedish general population					At-risk Norwegian youths^c^					European norm data^d^				
	*n*	*M*	SD	*n*	*M*	SD	*P*	Cohen’s *d*	*n*	*M*	SD	*p*	Cohen’s *d*	*n*	*M*	SD	*p*	Cohen’s *d*
General QoL	298	47.99	11.82	3283^a^	52.0	10.12	**< 0.001**	−** 0.36**						14932	48.51	9.28	.341	− 0.05
Physical wellbeing	298	46.30	13.21	202^b^	48.8	9.21	**0.020**	− **0.22**	246	47.05	10.55	.471	− 0.06	15239	48.57	9.64	< **.001**	− **0.20**
Psychological wellbeing	297	49.33	13.70	202^b^	53.4	10.93	**0.001**	−** 0.33**	246	49.02	11.22	.776	0.02	15323	48.83	9.78	.387	0.04
Parent relations and autonomy	297	52.80	13.01	202^b^	55.1	9.88	**0.034**	−** 0.20**	246	50.62	11.51	**.041**	**0.18**	15135	49.41	9.81	< **.001**	**0.29**
Peers and social support	296	50.22	11.63	202^b^	54.1	8.17	**< 0.001**	−** 0.39**	246	50.32	11.68	.921	− 0.01	15372	49.62	9.96	.306	0.06
School environment	298	48.35	11.98	202^b^	55.8	9.60	**< 0.001**	−** 0.69**	246	51.05	11.76	**.009**	−** 0.23**	15255	48.44	9.41	0.871	− 0.01

^a^Swedish youths from the general population aged 12–18 [35]

^b^A random population sample of Swedish youths aged 11–16 [37]

^c^Norwegian children with ill or substance abusing parents, aged 8–17, the article did not include general QoL scores [38]

^d^European norm data from the construction and validation of the KIDSCREEN instruments. Youth aged 12–18 from 13 European countries [28]

The other youth samples are used as the reference group in all comparisons, meaning that our sample has a lower score than the comparison group when cohen’s *d* is negative

Significant differences are marked in boldface

### Predictors of general QoL

Male gender and younger age predicted higher general QoL in all steps of the regression analyses (Table 3). Living in kinship care was predictive of higher general QoL compared to living in nonkin care in step two (*B* = 5.15, 95% CI [0.79, 9.51], *p* = 0.022), but this relationship was not significant when adjusting for child factors in step 3 (*B* = 3.32, 95% CI [− 1.17, 7.80], *p* = 0.143). Prosocial behavior was predictive of higher general QoL (*B* = 1.34, 95% CI [0.36, 2.32], *p* = 0.009). The full model explained 33% of the variance in general QoL.

**Table 3:** Associations between general quality of life (QoL) and contextual and child factors, adjusted for gender and age (*N* = 525)

	General QoL		
	_adj_*R*^2^	*B*	95% CI
Step 1: covariates	0.17		
Gender^a^		**7.40**	**[4.99, 9.82]**
Age (years)		**− 1.55**	**[− 2.14, − 0.96]**
Step 2: added contextual factors	0.21		
Gender^a^		**7.96**	**[5.48, 10.44]**
Age (years)		**− 1.48**	**[− 2.08, − 0.87]**
Maltreatment^b,d^		**− **0.12	[**− **2.18, 1.94]
Service contact^d^		**− **0.93	[**− **3.37, 1.50]
Type of placement^c^		**5.15**	**[0.79, 9.51]**
Years in current foster home		0.06	[**− **0.27, 0.38]
Step 3: added child factors	0.33		
Gender^a^		**7.71**	**[5.06, 10.36]**
Age (years)		**− 1.33**	**[− 1.94, − 0.73]**
Maltreatment^b,d^		0.20	[**− **1.78, 2.18]
Service contact^d^		0.28	[**− **2.64, 3.20]
Type of placement^c^		3.32	[**− **1.17, 7.80]
Years in current foster home		**− **0.17	[**− **0.55, 0.20]
Total difficulties^d^		**− **0.30	[-0.80, 0.20]
Prosocial behavior^d^		**1.34**	**[0.36, 2.32]**
Functional impairment^d^		0.34	[**− **0.64, 1.32]

_*adj*_*R*^*2*^ Adjusted *R* squared, *B* beta values (unstandardized coefficient), *CI* confidence interval

^a^Girls are the reference group

^b^A sum score of four maltreatment items (range 0–4)

^c^Nonkinship care is the reference group

^d^Variable was measured at T1

Significant associations are marked in boldface

### Predictors of the five dimensions of QoL

For all five QoL dimensions, male gender and younger age predicted higher QoL in all steps of the analyses (Table 4). More maltreatment experiences (*B* = 2.23, 95% CI [0.09, 4.37], *p* = 0.042), kinship care (*B* = 4.82, 95% CI [0.11, 9.52], *p* = 0.045), and more prosocial behavior (*B* = 1.53, 95% CI [0.40, 2.66], *p* = 0.010) predicted higher physical wellbeing. More prosocial behavior was also predictive of higher psychological wellbeing (*B* = 1.39, 95% CI [0.04, 2.73], *p* = 0.044). Living in kinship care was predictive of higher scores on the parent relations and autonomy dimension compared to living in nonkin care (*B* = 6.14, 95% CI [1.11, 11.17], *p* = 0.018). The full model ranged from explaining 40% of the variance in physical wellbeing to 12% of the variance in the social support and peers dimension.

**Table 4:** Associations between the five dimensions of quality of life (QoL) and contextual and child factors, adjusted for gender and age (*N* = 525)

	Physical wellbeing			Psychological wellbeing			Parent relations and autonomy			Social support and peers			School environment		
	_adj_*R*^2^	*B*	95% CI	_adj_*R*^2^	*B*	95% CI	_adj_*R*^2^	*B*	95% CI	_adj_*R*^2^	*B*	95% CI	_adj_*R*^2^	*B*	95% CI
Step 1: covariates	0.22			0.14			0.08			0.07			0.09		
Gender^a^		**7.81**	**[5.00, 10.61]**		**7.74**	**[4.87, 10.61]**		**6.39**	**[3.33, 9.44]**		**4.27**	**[1.62, 6.92]**		**4.00**	**[1.24, 6.76]**
Age (years)		**− 2.36**	**[− 3.03, − 1.69]**		**− 1.61**	**[− 2.29, − 0.94]**		**− 0.83**	**[− 1.52, − 0.14]**		**− 1.04**	**[− 1.66, − 0.41]**		**− 1.50**	**[− 2.15, − 0.84]**
Step 2: added contextual factors	0.29			0.18			0.17			0.09			0.13		
Gender^a^		**7.68**	**[4.55, 10.82]**		**8.41**	**[5.19, 11.64]**		**7.12**	**[3.88, 10.37]**		**4.35**	**[1.60, 7.09]**		**4.51**	**[1.45, 7.58]**
Age (years)		**− 2.48**	**[− 3.18, − 1.78]**		**− 1.61**	**[− 2.32, − 0.89]**		**− 0.84**	**[− 1.53, − 0.14]**		**− 0.98**	**[− 1.66, − 0.30]**		**− 1.44**	**[− 2.12, − 0.78]**
Maltreatment^b, d^		2.03	[− 0.02, 4.08]		0.08	[− 2.28, 2.43]		1.05	[− 1.28, 3.38]		− 0.29	[− 2.34, 1.76]		− 1.08	[− 2.54, 0.39]
Service contact^d^		0.13	[− 2.25, 2.51]		− 2.04	[− 4.95, 0.88]		− 2.17	[− 5.50, 1.16]		0.66	[− 1.87, 3.19]		− 0.38	[− 2.75, 1.98]
Type of placement^c^		**5.83**	**[1.86, 9.80]**		3.41	[− 0.47, 7.30]		**7.21**	**[2.35, 12.08]**		2.16	[− 1.82, 6.15]		3.02	[− 0.99, 7.03,]
Years in current foster home		0.12	[− 0.22, 0.47]		0.21	[− 0.19, 0.60]		0.19	[− 0.22, 0.59]		− 0.07	[− 0.47, 0.35]		0.15	[− 0.23, 0.53]
Step 3: added child factors	0.40			0.27			0.21			0.12			0.20		
Gender^a^		**7.15**	**[3.71, 10.58]**		**7.90**	**[4.58, 11.22]**		**7.07**	**[3.86, 10.28]**		**4.19**	**[1.37, 7.01]**		**4.24**	**[0.97, 7.50]**
Age (years)		**− 2.42**	**[− 3.15, − 1.68]**		**− 1.51**	**[− 2.24, − 0.78]**		**− 0.72**	**[− 1.43, − 0.00]**		**− 0.93**	**[− 1.63, − 0.23]**		**− 1.31**	**[− 1.97, − 0.64]**
Maltreatment^b, d^		**2.23**	**[0.09, 4.37]**		0.33	[− 2.05, 2.70]		1.17	[− 1.23, 3.56]		− 0.23	[− 2.29, 1.83]		− 0.89	[− 2.43, 0.65]
Service contact^d^		0.25	[− 2.52, 3.02]		− 1.30	[− 4.38, 1.78]		− 1.20	[− 4.81, 2.41]		0.92	[− 1.87, 3.71]		0.62	[− 1.88, 3.12]
Type of placement^c^		**4.82**	**[0.11, 9.52]**		2.03	[− 2.42, 6.47]		**6.14**	**[1.11, 11.17]**		1.58	[− 2.65, 5.80]		1.70	[− 2.53, 5.94]
Years in current foster home		− 0.15	[− 0.54, 0.24]		− 0.03	[− 0.43, 0.37]		0.12	[− 0.33, 0.56]		− 0.14	[− 0.53, 0.24]		− 0.01	[− 0.37, 0.34]
Total difficulties^d^		− 0.24	[− 0.78, 0.31]		− 0.31	[− 0.79, 0.17]		− 0.03	[− 0.57, 0.51]		− 0.07	[− 0.64, 0.49]		− 0.15	[− 0.57, 0.26]
Prosocial behavior^d^		**1.53**	**[0.40, 2.66]**		**1.39**	**[0.04, 2.73]**		0.58	[− 0.68, 1.85]		0.46	[− 0.64, 1.56]		1.14	[− 0.02, 2.29]
Functional impairment^d^		1.35	[− 0.11, 2.82]		0.92	[− 0.42, 2.27]		− 0.50	[− 1.77, 0.78]		0.22	[− 0.99, 1.44]		0.13	[− 0.96, 1.21]

_adj_*R*^2^ Adjusted *R* squared, *B* Beta values (unstandardized coefficient), *CI* Confidence interval

^a^Girls are the reference group

^b^A sum score of four maltreatment items (range 0–4)

^c^Nonkinship care is the reference group

^d^Variable was measured at T1

Significant associations are marked in boldface

## Discussion

The youths in foster care had lower general QoL and lower QoL across all dimensions than Swedish youths in the general population. However, compared to at-risk Norwegian youths and European norm data, the scores were similar on most dimensions. To our knowledge, this is the first prospective study to investigate the predictors of QoL among youths in foster care. Male gender, younger age, living in kinship care and more prosocial behavior five years earlier predicted higher QoL.

Our finding that youths in foster care had lower QoL than Swedish youths from the general population [32, 34] is in line with findings from Australia, where youths in foster care had lower HRQoL on most dimensions compared to the general population [7]. However, this contrast to a Serbian study, which observed no differences in HRQoL between youths in foster care and the general population [10]. The youths in our sample had similar QoL scores on most dimensions compared to European norm data. Sizeable differences in general QoL are observed between countries [32] and because Scandinavia has better health status and higher subjective wellbeing than most European countries [41], it seems plausible that Scandinavian youths will have higher QoL levels, as indicated by the high scores in the Swedish norm data.

We found that youths in foster care had lower physical wellbeing than the Swedish general population sample [34] and the European norm data [25], but similar levels to the at-risk Norwegian youths [35]. These findings imply that physical wellbeing and health are important to assess and target in interventions for at-risk youths. Our sample reported lower scores on the school environment dimension compared to the at-risk Norwegian youths, which might be a consequence of youths in foster care changing schools more often than other youths [42]. Youths in foster care reported the highest scores on the parent relations and autonomy dimension. These scores were lower than the scores from the Swedish general population sample but higher than the scores from European norm data and at-risk Norwegian youths. These findings suggest that despite their often detrimental care experiences, youths moved into adequate care conditions often form good relationships with their new caregivers. The effect sizes for the differences found between the compared youth groups ranged from 0.18 to 0.69, which are considered as small to medium according to Cohen [36]. However, even small differences may have substantial impact when they affect many people, as is the case for youths in foster care.

Male gender and younger age predicted higher QoL, which is in line with findings from the general population [16, 17] and from youths in care [10, 14]. While girls in the general European population reported higher scores on the peers and social support and school environment dimensions compared to boys [17], our results showed that girls had lower QoL across all dimensions. This might indicate that girls are especially vulnerable to the stressors of preplacement maltreatment and moving into foster care, and may need extra support to facilitate a positive development.

Living in kinship care predicted higher general QoL compared to living in nonkinship care, but only prior to controlling for the child factors. This might indicate that youths in kinship care report higher general QoL because of better mental health. This is in line with findings from Winokur et al. [24] that children in kinship care had higher wellbeing and fewer mental health disorders compared to children in nonkinship care. However, living in kinship care was predictive of higher physical wellbeing and higher scores on the parent relations and autonomy dimension compared to living in nonkinship care, even after adjusting for child factors, indicating that youths in kinship care have better physical health and better relations with caregivers. One might speculate that the CWS more often places youths with good health and good relations with their extended family in kinship care; alternatively, when the contact between youths and kinship caregivers is of high quality, this placement form supports contact with the biological family and their local community, which could lead to positive outcomes.

In contrast to previous findings that exposure to family violence was associated with lower HRQoL [23], we found that previous maltreatment predicted higher physical wellbeing. However, the effect was small and only present when controlling for the child factors; thus, further research on the relation between maltreatment and QoL among youths in foster care is warranted.

Neither years in the current foster home nor previous service contact was predictive of QoL, which contrasts to findings that youths with longer stays in the same placement reported higher subjective wellbeing [14]. However, the youths in our sample had lived seven years in their current foster home on average, which may limit our opportunity to discover possible negative effects of short stays and frequent moves on QoL. Moreover, mental health and functional impairment five years earlier did not predict QoL, indicating that childhood mental health problems do not necessarily lead to poor QoL among adolescents in foster care. This result was surprising, as studies have found associations between mental health and QoL [18] and that youths in foster care showed stable trajectories of mental health [43]. Our findings might be a consequence of youths receiving effective mental health services and/or positive development processes in the foster home. Prosocial behavior five years earlier predicted general QoL, physical wellbeing, and psychological wellbeing, indicating that building social skills among youths in foster care might be one way to enhance future QoL.

The full model of predictors explained 33% of the variance in general QoL; gender and age contributed to roughly half of the explained variance, indicating that these characteristics are important determinants of QoL. The explained variance varied between the QoL dimensions, with the included predictors having the greatest effect on physical wellbeing (40%) and the weakest effect on the social support and peers dimension (12%).

## Strengths and limitations

As this study used a QoL instrument with good cross-cultural validity [25], we have been able to compare QoL among youths in foster care to QoL in other youth populations. We have a fairly large sample of high-risk youths that are difficult to recruit and challenging to follow over time due to instability in their living arrangements. Consequently, a limitation of our study is that we have missing data between T1 and T2 that is mainly due to changes in the youths living arrangements making them ineligible for recruitment at T2 (e.g., adoption, moved within the last six months, moved to an institution or reunited with biological parents). The response rate of invited youths at T2 was somewhat low (41.9%), which could influence the generalizability of our results. However, there were no differences in baseline measures for families lost to follow-up, and we have no reason to assume that missing data were related to QoL. Furthermore, we used multiple imputation to handle the missing data, which is preferable over listwise and pairwise deletion, as it results in more statistical power, gives unbiased results when data are missing at random and less biased results than other methods when data are not missing at random [44].

In our study maltreatment was reported by caseworkers, which could influence the accuracy of the measure, as caseworkers do not have full information about children’s experiences. We considered to include the SDQ sub dimensions externalization and internalization problems as predictors in our analyses, but as these dimensions where highly correlated to total difficulties and to each other (data not shown) we only included total difficulties in the final analyses. We had no information on factors such as intelligence and socio economic status and future studies of QoL among youths in foster care ought to include such variables.

## Conclusions

Youths in foster care had lower QoL than Scandinavian youths in general, indicating that these youths need more support to enhance their QoL. The relatively high scores on the parent relations and autonomy dimension implies that these youths have supportive relationships with their carers. Our finding of higher QoL among boys and among younger youth suggests that adolescent girls might need extra support to have good lives. Furthermore, higher physical wellbeing and better carer-relations among youths in kinship care lend support to the ongoing preference for kinship placements when the extended family can provide adequate care. Last, our results indicate that it is important to build and strengthen relational resources among children who have experienced detrimental care conditions.

## Electronic supplementary material

Below is the link to the electronic supplementary material.

Supplementary file1 (DOCX 37 kb)

Supplementary file2 (DOCX 15 kb)

## Data Availability

Access to data is restricted by Norwegian law on medical and health related research. Information about the data and analysis is available from corresponding author Marit Larsen on request.

## Code availability

Code is available from Marit Larsen upon request.
