# Supplementary material for: Predictors of quality of life among youths in foster care—a 5-year prospective follow-up study
Source: Qual Life Res. 2020 Sep 24;30(2):543–54. doi: 10.1007/s11136-020-02641-z (PMC7886817; doi:10.1007/s11136-020-02641-z)
Supplement: Supplementary file 1 — Supplementary file1 (DOCX 37 kb) [file 11136_2020_2641_MOESM1_ESM.docx]

| Supplementary Table 1  *Predictors of general quality of life (QoL). Comparisons of results using three different methods of handling the missing data.* | | | | | | | | | | | | | | | |
| --- | --- | --- | --- | --- | --- | --- | --- | --- | --- | --- | --- | --- | --- | --- | --- |
|  | | QoL (FIML^e^ - cases with missing on IVs excluded) | | | |  | QoL (FIML^e^- all cases included) | | | |  |  | QoL (Multiple imputations) | | |
|  | | n | R^2^ | B | 95% CI |  | n | R^2^ | B | 95% CI |  | n | R^2^ | B | 95% CI |
| Step 1: covariates | | 523 | 0.17 |  |  |  | 525 | 0.17 |  |  |  | 525 | 0.17 |  |  |
|  | Gender^a^ |  |  | **7.27** | **[4.81, 9.73]** |  |  |  | **7.25** | **[4.80, 9.70]** |  |  |  | **7.40** | **[4.99, 9.82]** |
|  | Age (years) |  |  | **-1.50** | **[-2.10, -0.90]** |  |  |  | **-1.50** | **[-2.10, -0.90]** |  |  |  | **-1.55** | **[-2.14, -0.96]** |
| Step 2: added contextual predictors | | 111 | 0.24 |  |  |  | 525 | 0.20 |  |  |  | 525 | 0.22 |  |  |
|  | Gender^a^ |  |  | **8.29** | **[3.76, 12.83]** |  |  |  | **7.85** | **[5.24, 10.46]** |  |  |  | **7.96** | **[5.48, 10.44,]** |
|  | Age (years) |  |  | **-1.29** | **[-2.41, -0.17]** |  |  |  | **-1.40** | **[-2.06, -0.75]** |  |  |  | **-1.48** | **[-2.08, -0.87]** |
|  | Maltreatment^b, d^ |  |  | -0.69 | [-2.82, 1.44] |  |  |  | -0.27 | [-2.66, 2.13] |  |  |  | -0.12 | [-2.18, 1.94] |
|  | Service contact^d^ |  |  | -1.39 | [-4.02, 1.25] |  |  |  | -0.78 | [-3.58, 2.02] |  |  |  | -0.93 | [-3.37, 1.50] |
|  | Type of placement^c^ |  |  | 6.34 | [-0.54, 13.23] |  |  |  | **5.47** | **[1.62, 9.31]** |  |  |  | **5.15** | **[0.79, 9.51]** |
|  | Years in current foster home |  |  | -0.01 | [-0.59, 0.58] |  |  |  | -0.00 | [-0.36, 0.36] |  |  |  | 0.06 | [-0.27, 0.38] |
| Step 3: added child factors | | 82 | 0.28 |  |  |  | 525 | 0.31 |  |  |  | 525 | 0.34 |  |  |
|  | Gender^a^ |  |  | **5.76** | **[0.38, 11.14]** |  |  |  | **7.29** | **[4.63, 9.96]** |  |  |  | **7.71** | **[5.06, 10.36]** |
|  | Age (years) |  |  | **-1.24** | **[-2.47, -0.02]** |  |  |  | **-1.29** | **[-1.96, -0.62]** |  |  |  | **-1.33** | **[-1.94, -0.73]** |
|  | Maltreatment^b, d^ |  |  | 0.48 | [-2.24, 3.19] |  |  |  | 0.26 | [-2.04, 2.55] |  |  |  | 0.20 | [-1.78, 2.18] |
|  | Service contact^d^ |  |  | 0.50 | [-2.91, 3.90] |  |  |  | 1.00 | [-1.93, 3.93] |  |  |  | 0.28 | [-2.64, 3.20] |
|  | Type of placement^c^ |  |  | 4.18 | [-3.55, 11.90] |  |  |  | 3.28 | [-0.91, 7.48] |  |  |  | 3.32 | [-1.17, 7.80] |
|  | Years in current foster home |  |  | -0.42 | [-1.14, 0.30] |  |  |  | -0.25 | [-0.64, 0.13] |  |  |  | -0.17 | [-0.55, 0.20] |
|  | Total difficulties^d^ |  |  | -0.09 | [-0.60, 0.42] |  |  |  | -0.17 | [-0.62, 0.28] |  |  |  | -0.30 | [-0.80, 0.20] |
|  | Prosocial behavior^d^ |  |  | 1.33 | [-0.10, 2.76] |  |  |  | **1.66** | **[0.47, 2.85]** |  |  |  | **1.34** | **[0.36, 2.32]** |
|  | Functional impairment^d^ |  |  | 0.03 | [-1.22, 1.28] |  |  |  | 0.27 | [-0.95, 1.50] |  |  |  | 0.34 | [-0.64, 1.32] |
| Note: R^2^ - R squared. B - beta values (unstandardized coefficient). CI - Confidence interval. ^a^ Girls are the reference group. ^b^ A sum score of four maltreatment items (range 0 – 4). ^c^ Nonkinship care is the reference group. ^d^ Variable was measured at T1. ^e^ Full information maximum likelihood  Significant associations are marked in **boldface**. | | | | | | | | | | | | | | | |

| Supplementary Table 2  *Predictors of the five dimensions of quality of life (QoL; N = 525). Comparisons of results using full information maximum likelihood (FIML; all cases included) versus multiple imputations (MI) to deal with missing data.* | | | | | | | | | | | | | | | |
| --- | --- | --- | --- | --- | --- | --- | --- | --- | --- | --- | --- | --- | --- | --- | --- |
|  | | Physical wellbeing | |  | Psychological wellbeing | |  | Parent relations & autonomy | |  | Social Support & peers | |  | School environment | |
|  |  | B (FIML) | B (MI) |  | B (FIML) | B (MI) |  | B (FIML) | B (MI) |  | B (FIML) | B (MI) |  | B (FIML) | B (MI) |
| Step 1: covariates | |  |  |  |  |  |  |  |  |  |  |  |  |  |  |
|  | Gender^a^ | **8.00** | **7.81** |  | **7.99** | **7.74** |  | **6.48** | **6.39** |  | **4.42** | **4.27** |  | **3.98** | **4.00** |
|  | Age (years) | **-2.29** | **-2.36** |  | **-1.65** | **-1.61** |  | **-0.86** | **-0.83** |  | **-1.07** | **-1.04** |  | **-1.55** | **-1.50** |
| Step 2: added contextual predictors | |  |  |  |  |  |  |  |  |  |  |  |  |  |  |
|  | Gender^a^ | **7.68** | **7.68** |  | **8.82** | **8.41** |  | **7.06** | **7.12** |  | **4.55** | **4.35** |  | **4.49** | **4.51** |
|  | Age (years) | **-2.42** | **-2.48** |  | **-1.54** | **-1.61** |  | **-0.87** | **-0.84** |  | **-1.02** | **-0.98** |  | **-1.45** | **-1.44** |
|  | Maltreatment^b, d^ | 2.08 | 2.03 |  | -1.10 | 0.08 |  | 1.12 | 1.05 |  | -0.25 | -0.29 |  | -1.99 | -1.08 |
|  | Service contact^d^ | 0.73 | 0.13 |  | -1.76 | -2.04 |  | -1.72 | -2.17 |  | 0.60 | 0.66 |  | 0.18 | -0.38 |
|  | Type of placement^c^ | **5.75** | **5.83** |  | 3.17 | 3.41 |  | **7.12** | **7.21** |  | 2.88 | 2.16 |  | 2.95 | 3.02 |
|  | Years in current foster home | 0.10 | 0.12 |  | 0.10 | 0.21 |  | 0.20 | 0.19 |  | -0.30 | -0.07 |  | 0.07 | 0.15 |
| Step 3: added child factors | |  |  |  |  |  |  |  |  |  |  |  |  |  |  |
|  | Gender^a^ | **6.96** | **7.15** |  | **8.19** | **7.90** |  | **7.04** | **7.07** |  | **4.37** | **4.19** |  | **4.17** | **4.24** |
|  | Age (years) | **-2.42** | **-2.42** |  | **-1.51** | **-1.51** |  | -0.72 | **-0.72** |  | **-0.96** | **-0.93** |  | **-1.32** | **-1.31** |
|  | Maltreatment^b, d^ | **2.29** | **2.23** |  | -0.82 | 0.33 |  | 1.48 | 1.17 |  | -0.07 | -0.23 |  | -1.66 | -0.89 |
|  | Service contact^d^ | 1.43 | 0.25 |  | -0.59 | -1.30 |  | -0.66 | -1.20 |  | 1.34 | 0.92 |  | 1.37 | 0.62 |
|  | Type of placement^c^ | 4.06 | **4.82** |  | 1.72 | 2.03 |  | **5.64** | **6.14** |  | 1.84 | 1.58 |  | 1.06 | 1.70 |
|  | Years in current foster home | -0.24 | -0.15 |  | -0.16 | -0.03 |  | 0.10 | 0.12 |  | -0.18 | -0.14 |  | -0.14 | -0.01 |
|  | Total difficulties^d^ | -0.08 | -0.24 |  | -0.23 | -0.31 |  | 0.08 | -0.03 |  | 0.02 | -0.07 |  | -0.05 | -0.15 |
|  | Prosocial behavior^d^ | **1.95** | **1.53** |  | **1.59** | **1.39** |  | 0.91 | 0.58 |  | 0.84 | 0.46 |  | **1.48** | 1.14 |
|  | Functional impairment^d^ | 1.22 | 1.35 |  | 0.97 | 0.92 |  | -0.74 | -0.50 |  | 0.03 | 0.22 |  | 0.16 | 0.13 |
| Note: B - beta values (unstandardized coefficient). ^a^ Girls are the reference group. ^b^ A sum score of four maltreatment items (range 0 – 4). ^c^ Nonkinship care is the reference group.  ^d^ Variable was measured at T1  Significant associations are marked in **boldface**. | | | | | | | | | | | | | | | |

| Supplementary Table 3  *Pearson correlations between general quality of life (QoL), the five QoL dimensions and all predictors.* | | | | | | | | | | | | | | |
| --- | --- | --- | --- | --- | --- | --- | --- | --- | --- | --- | --- | --- | --- | --- |
|  | 1. | 2. | 3. | 4. | 5. | 6. | 7. | 8. | 9. | 10. | 11. | 12. | 13. | 14. |
| 1. General QoL |  |  |  |  |  |  |  |  |  |  |  |  |  |  |
| 2. Physical Wellbeing | .68** |  |  |  |  |  |  |  |  |  |  |  |  |  |
| 3. Psychological Wellbeing | .85** | .54** |  |  |  |  |  |  |  |  |  |  |  |  |
| 4. Parent relations & Autonomy | .76** | .43** | .60** |  |  |  |  |  |  |  |  |  |  |  |
| 5. Peers & Social Support | .64** | .47** | .60** | .46** |  |  |  |  |  |  |  |  |  |  |
| 6. School Environment | .78** | .47** | .69** | .50** | .50** |  |  |  |  |  |  |  |  |  |
| 7. Male gender^a^ | .32** | .32** | .31** | .26** | .20** | .18** |  |  |  |  |  |  |  |  |
| 8. Age (years) | -.28** | -.37** | -.26** | -.15** | -.20** | -.28** | -.05 |  |  |  |  |  |  |  |
| 9. Maltreatment^b, e^ | -.04 | .12 | -.11 | .07 | -.04 | -.23* | .07 | .08 |  |  |  |  |  |  |
| 10. Service contact^e^ | -.05 | .02 | -.09 | -.10 | .06 | .01 | .15* | .09 | .02 |  |  |  |  |  |
| 11. Kinship care^c^ | .17** | .15* | .10 | .19** | .09 | .11 | -.11*^d^ | -.06 | -.07 | -.09 |  |  |  |  |
| 12. Years in current foster home | -.03 | -.08 | .02 | .02 | -.04 | .03 | .03 | .21** | -.23** | .08 | -.04 |  |  |  |
| 13. Total difficulties^e^ | -.24* | -.03 | -.20 | -.16 | -.03 | -.19 | .02 | .08 | .11 | .37** | -.15 | -.07 |  |  |
| 14. Prosocial behavior^e^ | .30** | .18 | .31** | .19 | .10 | .32** | -.04 | -.06 | -.18* | -.22** | .21* | .24** | -.46** |  |
| 15. Functional impairment^e^ | -.13 | .08 | -.04 | -.18 | -.01 | -.11 | .07 | .13 | .04 | .41** | -.15 | .07 | .73** | -.35** |
| Note. * p < .05, ** p < .01. ^a^ Girls are the reference group. ^b^ A sum score of four maltreatment items (range 0 – 4). ^c^ Nonkinship care is the reference group. ^d^ The association between gender and type of foster care was tested with a chi-square test as both variables are binary. ^e^ Variable measured at T1. | | | | | | | | | | | | | | |
