# Supplementary material for: Predictors of quality of life among youths in foster care—a 5-year prospective follow-up study
Source: Qual Life Res. 2020 Sep 24;30(2):543–54. doi: 10.1007/s11136-020-02641-z (PMC7886817; doi:10.1007/s11136-020-02641-z)
Supplement: Supplementary file 2 — Supplementary file2 (DOCX 15 kb) [file 11136_2020_2641_MOESM2_ESM.docx]

| Appendix 1.  Overview of studies cited in the introduction, describing QoL or related measures | | | |
| --- | --- | --- | --- |
| Citation number | Article | Instrument used | Country |
| 5 | Ravens-Sieberer et al., 2014 | KIDSCREEN | 13 European countries |
| 6 | Greger, Myhre, Lydersen, & Jozefiak, 2016 | The Kinder Lebensqualitat Fragebogen (KINDL-R) | Norway |
| 7 | Carbone, Sawyer, Searle, & Robinson, 2007 | Child Health questionnaire (CHQ) | Australia |
| 8 | Bronsard et al., 2013 | Vécu et Santé Perçue de l’Adolescent (VSP-A questionnaire) | France |
| 9 | Seiler, Kohler, Ruf-Leuschner, & Landolt, 2016 | KIDSCREEN-27 | Chile |
| 10 | Damnjanović et al., 2012 | Pediatric Quality of life inventory (PedsQL) | Serbia |
| 11 | Jozefiak & Kayed, 2015 | The Kinder Lebensqualitat Fragebogen (KINDL-R) | Norway |
| 12 | Llosada-Gistau, Casas, & Montserrat, 2017 | The personal well-being index- school children (PWI-SC6) | Spain |
| 13 | Damnjanovic, Lakic, Stevanovic, & Jovanovic, 2011 | Pediatric Quality of life inventory (PedsQL) | Serbia |
| 14 | Llosada-Gistau, Casas, & Montserrat, 2019 | The personal well-being index- school children (PWI-SC6) and Student Life satisfaction scale (SLSS) | Spain |
| 15 | Li, Chng, & Chu, 2019 | Meta-analysis |  |
| 16 | Jernbro, Tindberg, Lucas, & Janson, 2015 | KIDSCREEN-10 | Sweden |
| 17 | Ravens-Sieberer et al., 2007 | KIDSCREEN-27 | 13 European countries |
| 18 | Gander et al., 2019 | Inventory of Life Quality in Children and Adolescents (ILC) | Switzerland |
| 19 | Jozefiak et al., 2017 | The Kinder Lebensqualitat Fragebogen (KINDL-R) | Norway |
| 20 | Weber, Jud, Landolt, & Goldbeck, 2017 | KIDSCREEN-10 | Germany |
| 21 | Lanier, Kohl, Raghavan, & Auslander, 2015 | Pediatric Quality of life inventory (PedsQL) | U.S. |
| 22 | Weber, Jud, & Landolt, 2016 | A systematic review |  |
| 23 | Beal et al., 2019 | Center for Disease Control Health-Related Quality of Life measure | U.S. |
| 24 | Winokur, Holtan, & Batchelder, 2014 | A Cochrane review |  |
| 26 | Ravens-Sieberer & Europe, 2006 | The KIDSCREEN questionnaires | 13 European countries |
